# The Tooth Brush as a Prophylactic

**Published:** 1876-03

**Authors:** 


### THE TOOTH BRUSH AS A PROPHYLACTIC.

BY M. C. S.

In the January number of the REGISTER, is an article read before the Merrimac Valley Dental Association with the above title, with which I take issue. The "opening" is good and as it is not on the subject at all, we have nothing to do with it.

As to the meaning of the word prophylactic, it simply means "I defend" or "preserve;" therefore as Dr. Robertson says, "a preventive of decay is a prophylactic," but (carrying it farther) all prophylactics are not preventives of decay; a tooth brush may be a prophylactic and not a preventative of decay, that is, may "defend" or "preserve" the teeth from other diseases.

But the gentleman has noticed "that persons who have taken the utmost possible care, for the cleanliness of their teeth by brushing, have suffered more from decay of their teeth, and have lost them sooner than many others who never use a tooth brush or took any sort of care for or about their teeth;" he then cites his own case as proof "that the tooth brush as a prophylactic, has but very little if any beneficial influence in any case, and nearly if not in all cases none at all." Were the conditions the same as to pre natal influences, hereditary predispositions, management after birth, food, drinks, climate and other prophylactic treatments? if so then the doctor has indeed proved the worthlessness of the tooth brush, and not only that, but has also proved that the tooth brush is an exciting cause of decay, for if these persons used a brush and the doctor did not, (their conditions influencing them being alike,) and their teeth decayed, while his did not, then the tooth brush is the cause. Behold! the tooth brush in a new role.

Do these cases cited prove anything? Yes, they prove that while the teeth of one person may be neglected and abused, the superiority of a natural or acquired, healthy and harmonious body, will resist their effects, and that one with hereditary or acquired, bodily infirmity, will, in spite of the best prophylactic treatment, succumb to them. Again because, "that in a vast majority of decayed teeth the caries is found in their approximal surfaces and in pits between the cusps of the bicuspid and multicuspid or molar teeth, where the tooth brush can not by any possibility reach," therefore it "has but very little if any beneficial influences," to those parts to which we can apply it, of course it has no "beneficial influence" to those parts we can not get at with it, this is not as the doctor put it, but is the natural sequence of his proposition carried further than he did it. I take it no one will claim the tooth brush a preventive of decay, only from the causes it removes, then what are the causes of decay a tooth brush removes. First, accumulation of food of all kinds, liable to decompositions, and by so doing change the original harmless articles into harmful ones; second, by keeping the teeth bright and clean, prevent the accumulation of salivary calculi; third, by brushing the gums, keep them in good condition, thereby preventing loose and spongy gums, and vitiated secretions.

With reference to brushing teeth where the saliva is acid, of course it is not expected that the brushing will either remove the acid condition or prevent decay, and in such cases the treatment should be constitutional principally, being honest enough to ascribe the decay that may be present to its true cause and not to the tooth brush, if we know what that is.

With reference to the dentist who said, "perfect cleanliness was a sure preventive of decay of the teeth," we say we hope his opinion is the exception and not the rule as we also hope Dr. Robertson's opinion of the tooth brush is.

